# Iodine concentration and content measured by dual-source computed tomography are correlated to thyroid hormone levels in euthyroid patients: a cross-sectional study in China

**DOI:** 10.1186/s12880-020-0411-8

**Published:** 2020-01-31

**Authors:** Zheng-Teng Li, Rui Zhai, Hong-Mei Liu, Min Wang, Dong-Mei Pan

**Affiliations:** Department of Radiology, Jining No.1 People’s Hospital, Jining, 272000 China

**Keywords:** Dual energy CT, Thyroid, Iodine concentration

## Abstract

**Background:**

The aim of this study was to investigate the correlation of the dual energy CT measured iodine concentration and total iodine content with blood measured thyroid parameters.

**Methods:**

Forty-three patients with normal thyroid function at our hospital from August 2017 to October 2019 were included in this retrospective study. Dual energy CT was used to scan the neck of thyroid patients. The mean iodine concentration and thyroid tissue volume were measured to calculate the total iodine content of the thyroid. Relevant tests of triiodothyronine (FT3), total triiodothyronine (TT3), total thyroxine (TT4), free thyroxine (FT4), and thyroid hormone (TSH) were conducted. The correlation of the thyroid mean iodine concentration and total iodine content with blood-measured thyroid function was analysed.

**Result:**

The total iodine content in the thyroid was positively correlated with FT3 but negatively correlated with TSH. The mean iodine concentration of the thyroid was positively correlated with both FT3 and TT3.

**Conclusion:**

The thyroid iodine content measured by dual energy CT can be used to determine the human iodine nutritional status and evaluate thyroid function, which will facilitate the diagnosis and treatment of thyroid diseases.

## Background

Iodine is one of the necessary trace elements for humans, and it is also the raw material for the synthesis of thyroid hormones. Iodine entering the human body concentrates in the thyroid gland. Both iodine deficiency and iodine excess can cause changes to the morphology and function of the thyroid [1, 2]. Therefore, the measurement of thyroid iodine is not only helpful to determine the human iodine nutritional status but also has a certain value for the evaluation of thyroid function. Previous studies measuring the iodine concentration in phantom have shown that dual energy CT (DECT) is highly accurate in measuring the iodine concentration [3, 4]. Shao Weiguang [5] and others proved that gemstone energy spectrum CT imaging made the measurement of iodine concentration more convenient, which can facilitate evaluations of thyroid function. Other studies have also confirmed that thyroid volume measured by CT is relatively accurate [6, 7]. We can measure the iodine concentration and thyroid volume by DECT and then multiply the two to obtain the total iodine content of the thyroid. Duong Duc Binh [8] et al. studied the relationship between the DECT-measured thyroid iodine concentration and the iodine uptake rate in patients with hyperthyroidism and concluded that an iodine uptake rate of 3 h was negatively correlated with the iodine concentration. At present, there are no studies on the relationship between the CT-measured thyroid iodine concentration, total iodine content and blood-measured thyroid function. In this investigation, we analysed the correlation between the DECT-measured thyroid mean iodine concentration, volume and total iodine content with blood-measured thyroid function to provide theoretical evidence for evaluating iodine nutritional status and thyroid function by DECT in the future.

## Methods

### Subjects

All patients who underwent neck DECT scanning from August 2017 to October 2019 were enrolled in this study. Inclusion criteria were as follows: patients who had a thyroid hormone test, which was performed 3 days within CT scanning. Exclusion criteria were as follows: (1) uneven thyroid density, with low-density lesions and calcification; (2) a history of thyroid surgery or thyroid artefacts seriously affected by the environment; (3) abnormal thyroid function; (4) recent treatment with thyroid preparations or iodine-containing drugs. A total of 43 patients (20 males and 23 females), aged 22–79 years, with an average age of 55.00 ± 13.67 years, were enrolled in the study. All selected cases were approved by the hospital ethics committee, and informed consent was signed by the patients before scanning.

### Computed tomography scanning and post-processing

CT scanning was performed using Siemens Definition Flash (SOMATOM. Definition flash; Siemens Healthcare, Forchheim, Germany). The scanning parameters were as follows: A tube voltage 100 kV, reference current 186 mAs; B tube voltage Sn140 kV, reference current 125 mAs, fusion coefficient 0.5, pitch 0.65, open CARE Dose 4D, Q30 (SAFIRE strength 3); and slice thickness/interval, 1.5/1.5 mm. Scanning ranged from the skull base to the thoracic entrance. The patient lay supine on the examination bed. The mandibular and shoulder positions were required in order to avoid the influence of clavicle artefacts. Instructions regarding breath holding and no swallowing were given to avoid breathing and swallowing artefacts.

### Measurement and data analysis

For measurement of the CT values and the iodine concentration of the thyroid glands, CT data was transferred to a standard post-processing workstation (Syngo Via workstation, Siemens Healthcare, Forchheim, Germany). The iodine map was obtained by choosing the “CT Dual Energy” mode. The iodine map image and the conventional 120 kVp images were generated from the low- voltage and high-voltage CT data sets with a slice thickness of 1.5 mm. The iodine concentrations and the CT values were measured from those images.

The slices for the ROI setting were carefully selected with use of the following criteria: (a) minimal beam hardening artefacts; (b) homogenous area; and (c) no nodular lesions. We manually marked the ROIs on the right and left lobes of the thyroid gland. The largest possible ROI (round or oval-shaped) was marked taking care not to include the margins of the thyroid tissue. The iodine concentration and CT value were measured three times. The average value of the ROI was set to 20 mm^2^. The left and right thyroid volumes, including the isthmus of the thyroid, were obtained through the outlined layer by layer, with the VOI Freehand option using the CT Bone Reading program. The mean iodine concentration and volume of thyroid tissue were measured, and the total iodine content (total iodine content = (mean iodine concentration × thyroid volume) was calculated.

### Detection of thyroid function

Fasting venous blood samples were collected in the morning, and serum free triiodothyronine (FT3), total triiodothyronine (TT3), free thyroxine (FT4), total thyroxine (TT4) and thyroid hormone (TSH) were detected by chemiluminescence immunoassay and analysed by gamma-ray radioimmunoassay. The instrument obtained the corresponding results.

### Statistical analysis

SPSS 20.0 software was used for statistical analysis. The correlation between mean iodine concentration, volume, total iodine content, age and thyroid function was analysed by Spearman correlation analysis. Statistical significance was defined at *p* < 0.05.

## Results

### The measurement of thyroid CT value, mean iodine concentration (Fig. 1a, b), thyroid volume (Fig. 2a, b) and total iodine content

The CT value of 43 adult thyroid tissues was 86.82 ± 20.56 HU, the average iodine concentration was 1.31 ± 0.46 mg/ml, the volume of thyroid was 12.87 ± 4.07 ml, and the total iodine content of thyroid was 16.70 ± 7.66 mg.

**Fig. 1:**
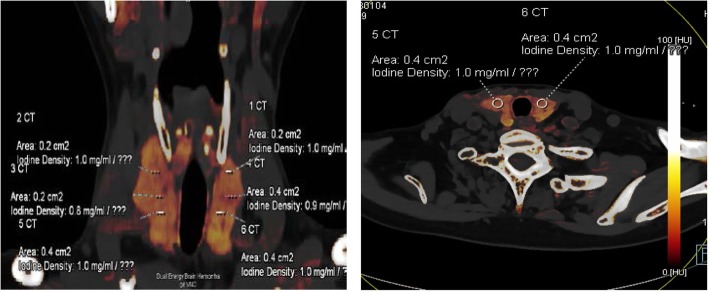
**a**, **b** Measurement of the iodine concentration in the left and right lobes of the thyroid by iodine mapping

**Fig. 2:**
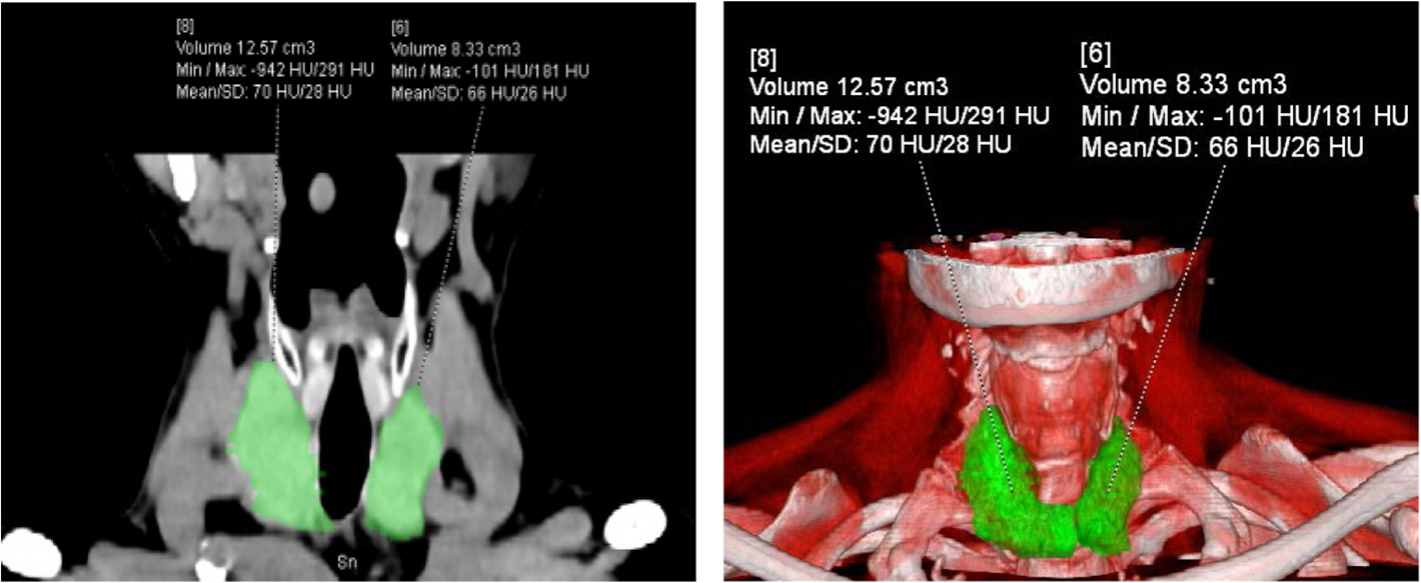
**a**, **b**. Measurement of the volume of the left and right lobes of the thyroid by depicting the area of interest

### Detection of thyroid function related indicators (Table 1)

A total of 42 cases were detected for the five thyroid function indices, including FT3, FT4, TSH, TT3 and TT4. In the other case, only FT3, FT4 and TSH were collected.

**Table 1:** Descriptive analysis of thyroid function

	FT3 (pmol/L)	FT4 (pmol/L)	TSH (μIU/ml)	TT3 (μIU/ml)	TT4 (pmol/L)
minimum	3.00	6.66	0.30	0.62	69.92
maximum	5.78	13.42	8.56	1.98	127.01
average	4.7230	10.3226	2.9060	1.4948	100.7574
standard deviation	0.68407	1.62327	2.09921	0.29166	11.86295
reference range	3.9–7.0	7.64–16.03	0.34–5.6	0.34–5.6	69.97–152.52
sample size	43	43	43	42	42

### Correlation analysis of age, mean iodine concentration, volume and thyroid iodine content with thyroid function index (Table 2)

**Fig. 3:**
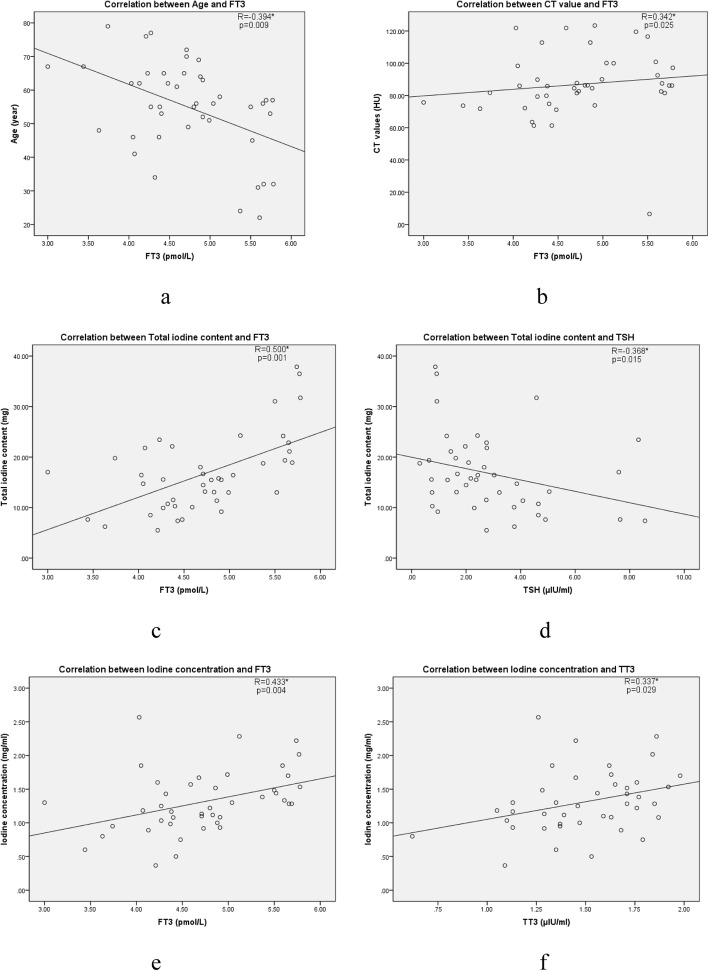
**a**. Correlation between age and FT3 distribution. **b**. CT value and FT3 distribution. **c**. Total iodine content and FT3 distribution. **d**. Total iodine content and TSH distribution. **e**. Iodine concentration and FT3 distribution. **f**. Iodine concentration and TT3 distribution

Correlation analysis of the results showed that age was negatively correlated with FT3, while CT value was positively correlated with FT3 (Fig. 3a, b). The total iodine content of the thyroid was positively correlated with FT3 (Fig. 3c) and negatively correlated with TSH (Fig. 3d). The thyroid iodine concentration was positively correlated with both FT3 and TT3 (Fig. 3e, f).

**Table 2:** Correlation analysis between the iodine level and thyroid function index (Spearman)

		FT3	TT3	FT4	TT4	TSH	Sex	Age	CT value
Total iodine content	R Value	0.500^*^	0.180	−0.088	0.194	−0.368^*^	− 0.432^*^	− 0.249	0.361^*^
	*p* Value	0.001	0.253	0.577	0.218	0.015	0.004	0.108	0.017
Iodine concentration	R Value	0.433^*^	0.337^*^	−0.132	0.122	−0.147	− 0.068	− 0.278	0.531^*^
	*p* Value	0.004	0.029	0.398	0.443	0.346	0.667	0.071	0.000
Age	R Value	−0.394^*^	−0.131	−0.008	0.224	0.227	0.117	1.000	−0.318^*^
	*p* Value	0.009	0.407	0.961	0.119	0.143	0.457	0.000	0.037
Volume	R Value	0.253	−0.107	−0.093	0.135	−0.273	− 0.590^*^	0.003	− 0.083
	*p* Value	0.102	0.502	0.555	0.393	0.076	0.000	0.987	0.599
CT value	R Value	0.342^*^	0.000	−0.040	0.066	−0.209	− 0.173	−0.318^*^	1.000
	*p* Value	0.025	1.000	0.800	0.677	0.178	0.268	0.037	0.000
Sample size	N	43	42	43	42	43	43	43	43

^*^p < 0.05

## Discussion

The thyroid gland is the main organ of iodine intake in the human body, and approximately 70–80% of the iodine in the human body is stored in the thyroid gland. Previous studies have shown that urinary iodine measurement can reflect the amount of human iodine intake. The correlation between urinary iodine and thyroid function has also been reported. The measurement of urinary iodine is affected by many factors, and it is difficult to reflect the iodine nutritional status of the body through this single test [9, 10]. In addition, studies on thyroid iodine by DECT have been limited to the measurement of the iodine concentration in the region of interest. To date, there is no simple, effective and non-invasive method to measure the thyroid iodine content.

DECT has two independent X-ray generation and detector systems. Two sets of spherical tubes can scan simultaneously. Because the attenuation coefficients of the same substance under different energy X-rays are different, DECT scanning technology can not only easily distinguish iodine from other substances but can also quantitatively calculate the iodine concentration in the region of interest with high accuracy. The mean iodine concentration and volume of the thyroid gland can be measured by DECT, and the total iodine content of the thyroid (total iodine content = thyroid mean iodine concentration × thyroid volume) can be calculated. The thyroid iodine concentration and total iodine content are relatively stable. The thyroid iodine concentration and total iodine content can be accurately measured by DECT and are highly consistent with the actual thyroid iodine concentration. Thus, DECT can be theoretically used as a reasonable method for the analysis of iodine nutritional status of the body.

The mean iodine concentration in the thyroid gland with normal thyroid function in the population was 1.31 ± 0.46 mg/ml, which was similar to that of 1.49 ± 0.41 mg/ml measured by gemstone energy spectrum CT imaging technology by Shao Weiguang [5] et al. In this study, we found no correlation between thyroid volume and thyroid function. This may be because the thyroid volume can be affected by many factors, such as height, weight, etc. [11, 12]. Serum FT3 showed a statistically significant (*p* < 0.01) decrease with age, which is consistent with the conclusion of the study of Harman SM [13], which reported that FT3 and T3 decreases with age. Our study also implied that ageing itself and non-thyroid diseases could lead to a decrease in serum TT3 concentration in the elderly, and this decrease may be due to the reduction of the peripheral transformation rate from TT4 to TT3. The mean iodine concentration of thyroid was positively correlated with both FT3 and TT3 (*p* < 0.05), and the total iodine content was positively correlated with FT3 (*p* < 0.01). This finding indicated that the mean iodine concentration of the thyroid could reflect the level of FT3 and TT3 in serum to some extent, which may be attributed to the reduction of thyroid iodine storage and iodine intake with ageing. As iodine is the main raw material for the synthesis of thyroid hormones, the reduction of stored iodine in the thyroid decreased the ability of the thyroid to synthesize and release FT3 and TT3. The total iodine content in the thyroid was negatively correlated with TSH (*p* < 0.05), which may be related to the decrease in serum FT3 and TT3 and the increase in serum TSH reactivity.

In this study, we concluded that thyroid CT values are positively correlated with iodine concentration and total iodine content. In theory, iodine is the main determinant of the thyroid CT value. Previous studies on iodine solution measurement have confirmed that there is a strong correlation between the CT value and the actual iodine concentration [14]. In this study, CT values were correlated with FT3 (*p* < 0.05) but were not correlated with TT3 and TSH. This may be explained by the fact that the measurement of thyroid tissue by the CT value can only partly reflect the iodine content of the thyroid, and it may also be affected by the density of thyroid tissue. Studies have shown that different thyroid disease tissues have different CT values, and in most cases, the CT values of thyroid disease tissues are lower than those of normal thyroid tissues [15, 16]. Therefore, compared with the CT value, the measurement of the iodine concentration and iodine content has more advantages in determining the pathological changes of thyroid tissue and iodine ion content in the thyroid. Thyroid diseases lead to the reduction of the iodine concentration and total iodine content. However, the degree of reduction will vary among different types of thyroid diseases. On the other hand, iodine content measurement by DECT can also be helpful for evaluating therapeutic effects in hyperthyroidism patients. Therefore, studies of the correlation of iodine concentration and iodine content with thyroid function in thyroid disease patients can be added to future studies to compensate for the deficiency of clinical practicability of this study.

### Limitations of this study

There are still some limitations of this study. Firstly, we had only 43 patients. The correlation study showed much variability in each comparison. Secondly, this study did not collect the iodine content and iodine uptake rate and other relevant parameters in the diet of patients and thus failed to employ a multivariate analysis to analyse the influencing factors for iodine content. Furthermore, the recent iodine intake status was not collected in this study, and we were thus unable to assess the impact of iodine intake on iodine content, which can be addressed in future research. Thirdly, we only studied the normal thyroid gland, not including dysfunctional thyroid glands. In the future, we will include a correlation study of the thyroid iodine content in patients with hyperthyroidism or hypothyroidism and a study of the changes in thyroid iodine content after treatment to obtain more valuable clinical results. Because ionizing radiation exposure co-occurs with CT examination, we will try to reduce the radiation dose to protect the patients. With the updating of equipment and technology, we can foresee that patients can have more accurate measurements with lower radiation doses in the future.

## Conclusion

The relationship of the thyroid iodine content and mean iodine concentration in adults to thyroid function was studied by DECT dual-energy scanning technology. We further demonstrated that DECT can be used to assess thyroid function and the iodine nutritional status of the body. Measurement of the thyroid iodine content and mean iodine concentration by DECT is simple, fast and effective and it will provide new methods and ideas for the future study of iodine-related thyroid diseases and the clinical diagnosis and treatment of thyroid diseases.

## Data Availability

The datasets analyzed during the current study are available from the corresponding author on reasonable request.

## Abbreviations

CT: Computed tomography
DECT: Dual energy computed tomography
FT3: Free triiodothyronine
FT4: Serum free thyroxine
HU: Hounsfield unit
ROI: Region of interest
TSH: Thyroid stimulating hormone
